# Editorial: Hybrid intelligent algorithms based learning, optimization, and application to autonomic control systems, volume II

**DOI:** 10.3389/fnbot.2023.1151473

**Published:** 2023-02-20

**Authors:** Yanzheng Zhu, Zhixiong Zhong

**Affiliations:** ^1^College of Electrical Engineering and Automation, Shandong University of Science and Technology, Qingdao, China; ^2^College of Computer and Control Engineering, Minjiang University, Fuzhou, China

**Keywords:** autonomous control systems, intelligent algorithms, optimization, robotics, switching control

With the advent of the age of artificial intelligence, a wealth of intelligent algorithms, such as genetic algorithm (Li et al., 2022), neural networks (Zou et al., 2020), and fuzzy logics (Li et al., 2019), etc., have been widely used in various fields during the past two decades. Specifically, thanks to the natures of the issue itself and sensor/actuator restrictions, continuous control is not appropriate and cannot be fulfilled, a possible selective in such circumstances is to incorporate logic-based decisions into the control law and perform switching among a family of controllers (Lin and Antsaklis, 2022). Nowadays, endless developments have been appeared to carry out the analysis and design of dynamic systems with typical hybrid dynamic characteristics. Unfortunately, the practical applications in the different fields, such as autonomous vehicles (Zhang et al., 2021), rehabilitation robots (Allen et al., 2022), and mechatronic systems (Liu et al., 2022), are of lack by using the existing hybrid intelligent algorithms to a great degree. Besides, it is not always easy to embed these hybrid intelligent algorithms to the application research and actual development of industrial installations.

More recently, various intelligent methodology and hybrid control technology have been developed for autonomic control systems in the literature (see Na et al., 2019; Allen et al., 2022; Che et al., 2022 and reference therein), which also have been applied to different scenarios in practice, including aerial vehicles (Kumar and Michael, 2012), permanent magnet synchronous machine (Egidio et al., 2022), and active magnetic bearing (Che et al., 2022), etc. Among them, it is desired that the sophisticated hybrid intelligent algorithms can be adapted to the learning-based optimization and control design to increase the autonomous performance of equipment. In addition, the exploration on the connection between the practical applications and the theoretical methodologies and technologies is very attractive with the aid of existing results based on learning and/or switching control approaches. This Research Topic presents a further collection about the learning and optimization of dynamic systems *via* hybrid intelligent algorithms, as well as their applications to the autonomic control systems.

Wang et al. propose a realistic module of traffic simulating to generate authentic traffic flow in the Test scenario, and to design a Hi-Fi truck model which is evaluated to imitate the actual truck response in the real world. Then, an AI planning module is established through a learning-based decision algorithm and a multi-mode trajectory planner, simultaneously considering the truck's restrictions, the road slope variations, and the environmental traffic flow. Finally, an automatic drive truck system is realized for road transport, which is the first attempt on the design of open-sourced full automatic drive truck system for logistic operation and volume-produce.

Consider a class of functional electrical stimulation (FES)-cycling system with unknown time-varying input delays, Tong and Zhu construct an Lyapunov-Krasovskii functional to investigate the stability and robustness of the presented system. By using the switching control approach, the considered rider-tricycle system is firstly decomposed into two subsystems. To avoid the chattering and destabilizing as high frequency switching occurs between FES and motor control, a novel average dwell time constraint is introduced to ensure the input-to-state stability (ISS) of the presented systems, and then the corresponding ISS condition is obtained for the augmented system. Finally, the performance of the designed state-feedback controller is testified *via* the simulation example under a wide range of time-varying delays, including the robustness even the time-varying input delays reach to 250 ms.

In reality, the application of micro-robots in medicine can break through the weaknesses and limitations of numerous conventional clinical approaches. In order to make the micro-robot smarter while passing through blood vessels, Huan et al. extract the skeleton of vascular images firstly. Then, a kind of skeleton-extraction-based A^*^algorithm is developed to determine an optimum route for the movement of micro-robots at a safe distance from the blood vessel wall. Moreover, the well-known gradient descent algorithm is borrowed to realize the smoothing of the planning paths, which results in a safe and smooth path of the micro-robots under the blood vessel environment.

Zhang et al. exploit a Multi-Layer Convolutional Neural Network (i.e., ResNet-18) and Long Short-Term Memory (LSTM) Networks model to perform the dynamic gesture recognition. A group of velocity-range Doppler images, which are transformed from the original signal, are generated as the input of the model. In particular, ResNet-18 is employed to extract the spatial features at a deeper level and to cope with the issue of gradient extinction/explosion, and LSTM is borrowed to extract temporal features and to address the issue of long-time dependence. Finally, the dynamic gesture recognition experiment is made on the Soli Dataset to implement the proposed model, and the degree of gesture recognition accuracy arrives at 92.55%.

To provide the real-time regolabile magnetic field distribution in its workspace through external programmable current suppliers, a novel quadrupole electromagnetic drive system is established by Ma et al. which is made up of four electromagnetic coils, each coil being energized *via* an independent DC power supplier. The system structure is constructed to accomplish an adjustable workspace and the parameters of the system are optimized *via* the parametric modeling approach and ANSYS. Moreover, a magnetic field map is created to promptly obtain the expected driving current from the needed magnetic flux density. Finally, experiments are set up for manipulation of micro-particles with the developed machinery.

## Author contributions

All authors listed have made a substantial, direct, and intellectual contribution to the work and approved it for publication.
